# Scoparone Improves Nonalcoholic Steatohepatitis Through Alleviating JNK/Sab Signaling Pathway-Mediated Mitochondrial Dysfunction

**DOI:** 10.3389/fphar.2022.863756

**Published:** 2022-05-03

**Authors:** Yuwei Jiang, Jiaoya Xu, Ping Huang, Lili Yang, Yang Liu, Yiping Li, Jue Wang, Haiyan Song, Peiyong Zheng

**Affiliations:** ^1^ Institute of Digestive Diseases, Longhua Hospital, Shanghai University of Traditional Chinese Medicine, Shanghai, China; ^2^ Department of Gout, Guanghua Hospital, Shanghai University of Traditional Chinese Medicine, Shanghai, China

**Keywords:** nonalcoholic steatohepatitis, mitochondrial dysfunction, lipotoxic injury, C-jun N-terminal kinase, SH3 domain-binding protein 5, scoparone

## Abstract

The activated c-Jun N-terminal kinase (JNK) specifically combined with SH3 domain-binding protein 5 (Sab) may mediate damage to the mitochondrial respiratory chain. Whether mitochondrial dysfunction induced by the JNK/Sab signaling pathway plays a pivotal role in the lipotoxic injury of nonalcoholic steatohepatitis (NASH) remains a lack of evidence. Scoparone, a natural compound from Traditional Chinese Medicine herbs, has the potential for liver protection and lipid metabolism regulation. However, the effect of scoparone on NASH induced by a high-fat diet (HFD) as well as its underlying mechanism remains to be elucidated. The HepG2 and Huh7 cells with/without Sab-knockdown induced by palmitic acid (PA) were used to determine the role of JNK/Sab signaling in mitochondrial dysfunction and cellular lipotoxic injury. To observe the effect of scoparone on the lipotoxic injured hepatocytes, different dose of scoparone together with PA was mixed into the culture medium of HepG2 and AML12 cells to incubate for 24 h. In addition, male C57BL/6J mice were fed with an HFD for 22 weeks to induce the NASH model and were treated with scoparone for another 8 weeks to investigate its effect on NASH. Molecules related to JNK/Sab signaling, mitochondrial function, and lipotoxic injury were detected in *in vitro* and/or *in vivo* experiments. The results showed that PA-induced activation of JNK/Sab signaling was blocked by Sab knockdown in hepatocytes, which improved mitochondrial damage, oxidative stress, hepatosteatosis, cell viability, and apoptosis. Scoparone demonstrated a similar effect on the PA-induced hepatocytes as Sab knockdown. For the NASH mice, treatment with scoparone also downregulated the activation of JNK/Sab signaling, improved histopathological changes of liver tissues including mitochondrial number and morphology, lipid peroxide content, hepatosteatosis and inflammation obviously, as well as decreased the serum level of lipid and transaminases. Taken together, this study confirms that activation of the JNK/Sab signaling pathway-induced mitochondrial dysfunction plays a crucial role in the development of NASH. Scoparone can improve the lipotoxic liver injury partially by suppressing this signaling pathway, making it a potential therapeutic compound for NASH.

## Introduction

Nonalcoholic fatty liver disease (NAFLD) is the most common chronic liver disease worldwide, which encompasses the spectrum from simple nonalcoholic fatty liver (NAFL), to nonalcoholic steatohepatitis (NASH), and liver cirrhosis (Cotter and Rinella, 2020). NASH is the progressive form of NAFLD, characterized by the presence of inflammation with or without fibrosis in addition to hepatic steatosis (Younossi et al., 2019). Previous studies have shown that the onset of NASH is triggered by lipotoxic liver injury (Machado and Diehl, 2016). Excessive lipid accumulation promotes insulin resistance, oxidative stress, mitochondrial dysfunction, and endoplasmic reticulum stress, resulting in cell apoptosis, inflammation, and fibrosis of liver tissues (Herbert and Alexander, 2010). However, up to now, its mechanism has not been completely clarified, and there is no effective treatment available for NASH (Patel and Siddiqui, 2019).

Mitochondria produce energy for eukaryotic cells through oxidative phosphorylation and electron transport (Vinten-Johansen, 2020). Recently, mounting evidence has revealed that mitochondrial dysfunction is closely involved in NASH development (Patterson et al., 2016). Excessive accumulation of free fatty acids in the liver could exacerbate reactive oxygen species (ROS) production during oxidation, which in turn suppresses the enzyme activities within the mitochondrial respiratory chain and results in mitochondrial dysfunction, thereby affecting energy metabolism and cell damage (Genova and Lenaz, 2014; Sunny et al., 2017). The mitochondrial membrane protein SH3 domain-binding protein 5 (Sab) is a scaffold protein located on the outer mitochondrial membrane. It can interact with the key kinase Bruton’s tyrosine kinase (BTK) and stress-activated protein kinase 3 (SAPK3) to regulate B cell growth and mitochondrial signal transcription (Tsukada, 1998; Wiltshire et al., 2002; Court et al., 2004). Sab activation was found up-regulated in Alzheimer’s disease, neonatal cerebral ischemic injury and liver injury, etc. (Wiltshire et al., 2002; Nijboer et al., 2013). c-Jun N-terminal kinase (JNK) is a member of the mitogen-activated protein kinase (MAPK) family. Its activation played a pivotal role in lipotoxic damage (Rockenfeller et al., 2010; Ibrahim and Gores, 2012). Studies have shown that during liver injury, JNK combined with Sab, can trigger the disruption of mitochondrial electron transport chain and promote ROS release, ultimately leading to the death of hepatocytes (Takeshita et al., 2013; Win et al., 2016). However, whether JNK/Sab signaling pathway induces lipotoxic liver injury by mediating mitochondrial dysfunction in NASH still lacks evidence.

Scoparone (Scop) is a natural compound from Traditional Chinese Medicine (TCM) herbs such as Artemisia scoparia Waldst. etKit and Artemisia capillaris Thunb., with the chemical name 6,7-dimethoxycoumarin (Jin et al., 2005; Yan et al., 2011). It has the function of relieving asthma and cough, anti-myocardial injury, liver protection, anti-tumor, and so on (Nawrot-Modranka et al., 2006; Kang et al., 2013; Fang et al., 2016; Wan et al., 2018). The pharmacological activities of scoparone include anti-inflammatory, antioxidant, anti-apoptotic, anti-fibrotic, and hypolipidemic effects. In recent years, mounting evidence has shown its therapeutic potential in various liver diseases, such as acute liver injury, alcohol-induced hepatotoxicity, NAFLD, and liver fibrosis (Hui W. Y. et al., 2020). In our preliminary experiment, scoparone was found able to improve PA-induced lipid deposition and lipotoxic injury of the hepatocyte. In addition, we also find that scoparone could inhibit fatty acid-induced JNK activation.

Therefore, this study aims to clarify the contribution of JNK/Sab signaling-mediated mitochondrial dysfunction to NASH, then to explore the role of scoparone against NASH and whether the JNK/Sab signaling pathway-mediated lipotoxic injury in hepatocytes is involved in its underlying mechanism through *in vivo* and *in vitro* experiments.

## Materials and Methods

### Cell Culture and Experimental Design for *in vitro* Experiment

Mouse hepatocyte AML12 and human hepatocarcinoma cell line HepG2, Huh7 were purchased from the Cell Biology Institute of Chinese Academy of Science (Shanghai, China) and cultured in DMEM with 10% FBS and 1% penicillin/streptomycin (Lonsera, Grand Island, United States) at 37°C in a humidified atmosphere containing 5% CO_2_.

To induce hepatic steatosis model, cells were incubated in DMEM containing 0.5 mM palmitic acid (PA) and 1% BSA (Sigma, Steinheim, Germany) for 24 h. The cells were treated with scoparone at different doses simultaneously. The cells cultured in the DMEM with 1% BSA were used as normal control. Scoparone was purchased from Shanghai Winherb Pharmaceutical Technology Development Co., Ltd. (Batch No: 190623; Purity ≥98%), and initially dissolved in 50 mM dimethyl sulfoxide (DMSO). The final concentrations of DMSO were kept below 0.1% in all culture conditions.

### Establishment of Sab Knockdown Cells

Three Sab-RNAi lentiviral vectors (sh Sab1, sh Sab2, and sh Sab3) were constructed by Genomeditech Co, Ltd (Shanghai, China) by using the vector pGMLV-SC5 RNAi-GFP. HepG2, Huh7, and AML12 cells were cultured in a 6-well plate (5 × 10^4^ cells/well) for 24 h, and were transfected with sh Sab lentivirus with a multiplicity of infection (MOI) of 10 to establish the stable Sab knockdown cell line. Cells transfected with empty-vector lentivirus were used as a scramble. Polybrene (2 μg/ml) was used to enhance transfection efficiency. After 72 h, RNA and protein of cells were extracted to detect Sab expression level to evaluate the transfection and knockdown efficiency.

### Cell Viability Assay

The cell viability was measured using the Cell Counting Kit-8 (CCK-8, Dojindo, Kumamoto, Japan). The cells were cultured in a 96-well plate (5 × 10^4^ cells/well), and treated with 0.5 mM PA for 24 h. Then, a 110 μl CCK-8 detection reagent (CCK-8 detection solution: DMEM = 1:10) was added and incubated for 4 h. The optical density (OD) of the cultures was detected at the absorbance of 450 nm by using Synergy H4 Hybrid Multi-Mode microplate reader (BioTeck, Winooski, United States).

### DAPI and Nile Red Double Staining of Cells

The cells were fixed with 4% paraformaldehyde and then stained using Nile Red (SIGMA, Steinheim, Germany) and DAPI (MP, Biomedicals, United States). The cell image was acquired by ImageXpress Microsystem High-content imaging system (Molecular Devices, LLC. San Jose, CA, United States). The cellular imaging analysis software MetaXpress Analysis (Molecular Devices) was used to run the quantitative analysis of the lipid content in cells.

### TMRM Assay and ROS Generation Determination

Tetramethyl rhodamine, methyl ester (TMRM) staining can quantify changes in mitochondrial membrane potential in living cells. It was used to monitor mitochondrial function. Cells were incubated with TMRM (Sigma, Steinheim, Germany) staining solution at 37°C for 20 min in the dark. Then TMRM was replaced with Hank’s solution, and finally, cells were viewed and photographed by ImageXpress Microsystem High-content imaging system (Molecular Devices).

Intracellular reactive oxygen species (ROS) generation level was determined by using the probe 2′,7′-dichlorodihydrofluorescein diacetate (DCFH-DA) (Beyotime Biotechnology, Shanghai, China). Briefly, cells were incubated with DCFH-DA in DMEM for 20 min. After washing twice with PBS, cells were observed and photographed under a fluorescence microscope (Olympus IX71, Tokyo, Japan).

### Experimental Design for *in vivo* Experiment

A total of 40 male C57BL/6J mice (6-week old) of SPF grade, were purchased from Shanghai Slack Laboratory Animal Co., Ltd. (license number SCXK (Shanghai) 2017-0005), and bred at room temperature (22°C) and relative humidity 50–70%. The mice were divided into a control group, a model group, and three scoparone intervention groups (low, medium, and high dose scoparone group), by using a completely random design according to their body weights (*n* = 8 for each group). The mice in the control group were fed a normal diet and the mice in the model group and intervention groups with a high-fat diet (HFD) (Research diet, Inc., New Brunswick, United States, D12492) for 30 weeks. From the 23rd week, mice of the low, medium, and high scoparone groups were fed with HFD supplemented with scoparone powder, at a dose of 25, 50, and 100 mg/kg body weight for 8 weeks. At the end of the experiment, the mice were fasted overnight, anesthetized, and sacrificed. The blood and liver tissues were collected for the subsequent experiments. The animal experiment procedure was approved by the Animal Experiment Ethics Committee of Longhua Hospital, Shanghai University of Traditional Chinese Medicine (No. LHERAW-190001). All animals were kept in compliance with the National Guideline for the Care and Use of Animals.

### Serum Biochemical Analysis and Detection of Serum TNF-α

The mouse serum was separated at 3,000 r/15 min, and the serum alanine aminotransferase (ALT), aspartate aminotransferase (AST), triglyceride (TG)、cholesterol (TC), high-density lipoprotein cholesterol (HDL), and low-density lipoprotein cholesterol (LDL) were measured by using ROCHE cobas 8,000 modular analyzer series and corresponding reagent kits (Basel, Switzerland). The serum TNF-α was determined by using an ELISA assay (Shanghai WestTang Bio-tech Co., Ltd) according to the manufacturer’s protocol.

### Measurement of TG and TC Content in Liver Tissues

Mouse live tissues (40 mg) were homogenized with 360 μl alcohol, then centrifuged for 15 min at 4°C, 2,500 rpm. The supernatant was measured based on the instructions of a TG or TC assay kit (Jiancheng Institute of Bio Engineering, Inc.) using the GPOPAP enzyme method. The absorbance was measured by Synergy H4 Hybrid Reader (BioTek, United States), and calculations of TG and TC content were performed according to the formula of the kit.

### Histopathology of Liver Tissues

The liver tissue was fixed with 4% paraformaldehyde, then dehydrated and embedded in paraffin and cut into 5 µm sections. The section was stained with hematoxylin-eosin (HE) solution (Yixin Biotechnology, Shanghai, China) according to a standard procedure. After mounting, the pathological changes of liver tissue were observed and photographed under a light microscope (Nikon ECLIPSE 50i, Tokyo, Japan).

### TUNEL Staining of Liver Tissues

The paraffin-embedded liver tissue sections were deparaffinized, hydrated, and permeabilized. A TUNEL apoptosis detection kit (*In Situ* Cell Death Detection Kit, Roche, Indianapolis, United States) was used to label the apoptotic cells with TMR red, and nuclei were counterstained with DAPI. The images of slides were scanned and analyzed by using ImageXpress MicroSystem and MetaXpress Analysis (Molecular Devices).

### The Observation of Hepatocyte Mitochondrion by Transmission Electron Microscope

The fresh liver tissues were fixed in 2% glutaraldehyde for 2 h, and the samples were then processed and photographed using HITACHI H-7650 transmission electron microscope in the Science and Technology Experiment Center of Shanghai University of Traditional Chinese Medicine.

### Immunohistochemistry of 4-HNE of Liver Tissues

The immunohistochemical experiment was performed to detect the level of 4-hydroxynonenal (4-HNE) in liver tissues. The paraffin-embedded liver sections were dewaxed, antigen-retrieved, and blocked, then incubated with a 4-HNE antibody (Alpha Diagnostic, Texas, United States) overnight. The secondary antibody and chromogenic reagent (Gene technology, Shanghai, China), were used to detect the positive stain. After being counterstained with hematoxylin, the section was photographed.

### Real-Time Quantitative Reverse Transcription-Polymerase Chain Reaction

The total RNA from liver tissue or cells was extracted with Trizol (Invitrogen, United States) reagent, reverse-transcribed with a reverse transcription kit (Applied Biosystems, Carlsbad, CA, United States). qRT-PCR was then performed with an SYBR Green PCR Mix kit (Accurate Biology, Changsha, China) with StepOnePlus Real-Time PCR System (Applied Biosystems). The expression level relative toβ-actin was calculated with the 2^−ΔΔct^ method. The gene sequence was verified on the Blast website, and the primers were synthesized by Shanghai Shinegene Biotechnology. The sequence of the primers was listed in Table 1.

**TABLE 1:** The sequences of primers used in PCR.

Gene	Sequence(5′-3′)
h β-actin	Forward: TCA​AGA​AAG​GGT​GTA​ACG​CAA​TA
	Reverse: CGA​CAG​GAT​GCA​GAA​GGA​GAT
h Sab	Forward: AGT​TCC​GCT​CTG​TTC​TGG​TTG
	Reverse: CCT​CTG​GAA​GTC​CTG​CGT​G
h PGC-1α	Forward: CAA​ATA​TCT​GAC​CAC​AAA​CGA​TG
	Reverse: AAG​TTG​TTG​TTG​GTT​TGG​CTT​GTA​AG
h NRF1	Forward: CCA​GTT​TAG​TGG​GTG​GTA​GG
	Reverse: CGG​GAG​CTT​TCA​AGA​CAT​TC
h TFAM	Forward: GTCACTGCCTCATCCACC
	Reverse: CCGCCCTATAAGCATCTT
m β-actin	Forward: GAG​ACC​TTC​AAC​ACC​CCA​GC
	Reverse: ATG​TCA​CGC​ACG​ATT​TCC​C
m PGC-1α	Forward: TGGCACGCAGCCCTATTC
	Reverse: GAG​GAT​CTA​CTG​CCT​GGG​GAC
m NRF1	Forward: TCC​CAG​AGA​TGC​TCA​AGT​ATT​CC
	Reverse: TTA​ACT​ATG​GTC​CGT​AAT​GCC​TG
m TFAM	Forward: GCA​TCC​CCT​CGT​CTA​TCA​GTC
	Reverse: TGT​GGA​AAA​TCG​AAG​GTA​TGA​AC
m IL-1β	Forward: GCT​TCA​GGC​AGG​CAG​TAT​CA
	Reverse: TGC​AGT​TGT​CTA​ATG​GGA​ACG
m TNF-α	Forward: CCC​TCC​AGA​AAA​GAC​ACC​ATG
	Reverse: CAC​CCC​GAA​GTT​CAG​TAG​ACA​G
h mtDNA	Forward: CCA​CTT​TCC​ACA​CAG​ACA​TCA
	Reverse: TGG​TTA​GGC​TGG​TGT​TAG​GG
m mtDNA	Forward: ACA​TCT​CGA​TGG​TAT​CGG​GTC
	Reverse: CCT​TAG​GTG​ATT​GGG​TTT​TGC

### Relative Copy Number of Mitochondrial DNA

The total DNA of liver tissue or cells was extracted by using a DNA extraction kit (Tiangen Biotech, Shanghai, China). The expression level of mitochondrial DNA (mtDNA) was detected by real-time PCR. The primer sequences, which are located at the D-loop region in mitochondrial DNA, were also listed in Table 1.

### Western Blot

The liver tissue and cell proteins were extracted with RIPA lysate (Beyotime Biotechnology), and the concentration was determined using the BCA method. The protein was separated by 10% acrylamide gel electrophoresis and transferred to the PVDF membrane (Millipore, Darmstadt, and Germany). TBST solution containing 5% skim milk (BD, Maryland, United States) was used to block the membrane for 1 h, followed by incubation with primary antibodies at 4°C overnight. Antibodies against P-JNK, JNK, P-Src, Src, P-SHP-1, SHP-1, Peroxisome proliferator-activated receptor-γ coactivator-1α (PGC-1α), and β-actin were purchased from Cell Signaling (Massachusetts, United States). Antibodies against Sab and Cleaved PARP were obtained from Proteintech (Wuhan, China) and ABclonal (Wuhan, China) respectively. The membrane was then incubated with the secondary antibodies at room temperature for 1 h and subsequently incubated with ECL luminescent substrate (Millipore, Billerica, United States). The signals were acquired using Tanon-5200 chemiluminescence image analysis system (Shanghai, China).

### Statistical Analysis

The measurement data were expressed as mean ± standard deviation (SD). All data were statistically analyzed using SPSS24.0 (SPSS Inc., Chicago, IL, United States) and Graphpad Prism 8.0 software (GraphPad Software Inc., San Diego CA, United States). Student *t-*test was used to compare the means of the two groups, and One-way analysis of variance (ANOVA) followed by Tukey’s post hoc comparison for three or more groups. *p* < 0.05 was considered statistically significant.

## Results

### Sab Knockdown Suppressed PA-Induced Activation of JNK/Sab Signaling Pathway in Hepatocytes

**FIGURE 1:**
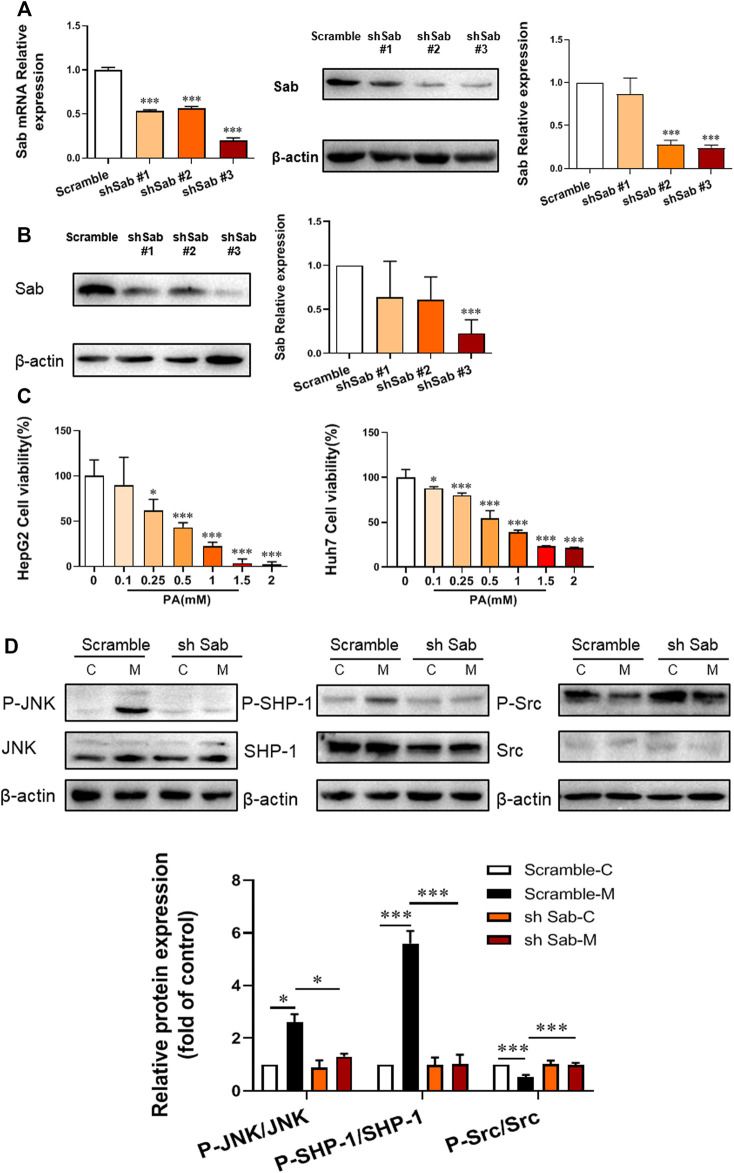
Sab knockdown inhibited PA-induced activation of JNK/Sab signaling pathway in hepatocytes. **(A)** The mRNA and protein expression level of Sab in HepG2 cells transfected with Sab shRNA lentivirus or scramble. ***p < 0.001 vs. Scramble. **(B)** The protein expression level of Sab in Huh7 cells with Sab shRNA lentivirus or scramble. ***p < 0.001 vs. Scramble. **(C)** The effect of different concentrations of PA on cell viability. *p < 0.05, ***p < 0.001 vs. PA 0 mM group. **(D)** The expression and activation level of JNK, SHP-1, and Src protein in HepG2 cells with PA induction (Model) or without (Control). *p < 0.05, ***p < 0.001. *n* = 3.

To identify the role of JNK/Sab signaling in the NASH mechanism, the key molecule Sab was knockdown in hepatocytes to block this pathway by using RNA interference. The expression of Sab mRNA and protein in hepatocytes transfected with Sab shRNA lentivirus or scramble was detected by qRT-PCR and Western blot. Figures 1A,B showed that sh Sab#3 lentiviral transfection had the most obvious inhibitory effect on Sab expression, with an inhibition rate of more than 80% in both HepG2 and Huh7 cell lines and around 60% in AML12 cells (Supplementary Figure S1A).

To imitate the lipotoxic liver injury of NASH *in vitro*, hepatocytes were incubated with PA (0.1, 0.25, 0.5, 1, 1.5, and 2 mM) for 24 h and the corresponding cell viability was measured by CCK8 assay. The cell viability was down-regulated by PA incubation in a dose-dependent manner. The cell survival rate was around 50% with 0.5 mM PA induction in HepG2 and Huh7 cells, which was thereby selected as the dose of PA for subsequent experiments (Figure 1C).

Figure 1D showed that PA increased the P-JNK/JNK and P-SHP-1/SHP-1 ratios of protein expression in HepG2 cells, whereas decreased P-Src/Src ratio. Knockdown of Sab prevented the PA-induced activation of JNK/Sab signaling, demonstrated by the significantly reduced P-JNK/JNK and P-SHP-1/SHP-1 ratios, and increased P-Src/Src value.

### PA-Induced Mitochondrial Dysfunction and Lipotoxic Injury of Hepatocytes Were Reversed by Suppression of the JNK/Sab Signaling Pathway

**FIGURE 2:**
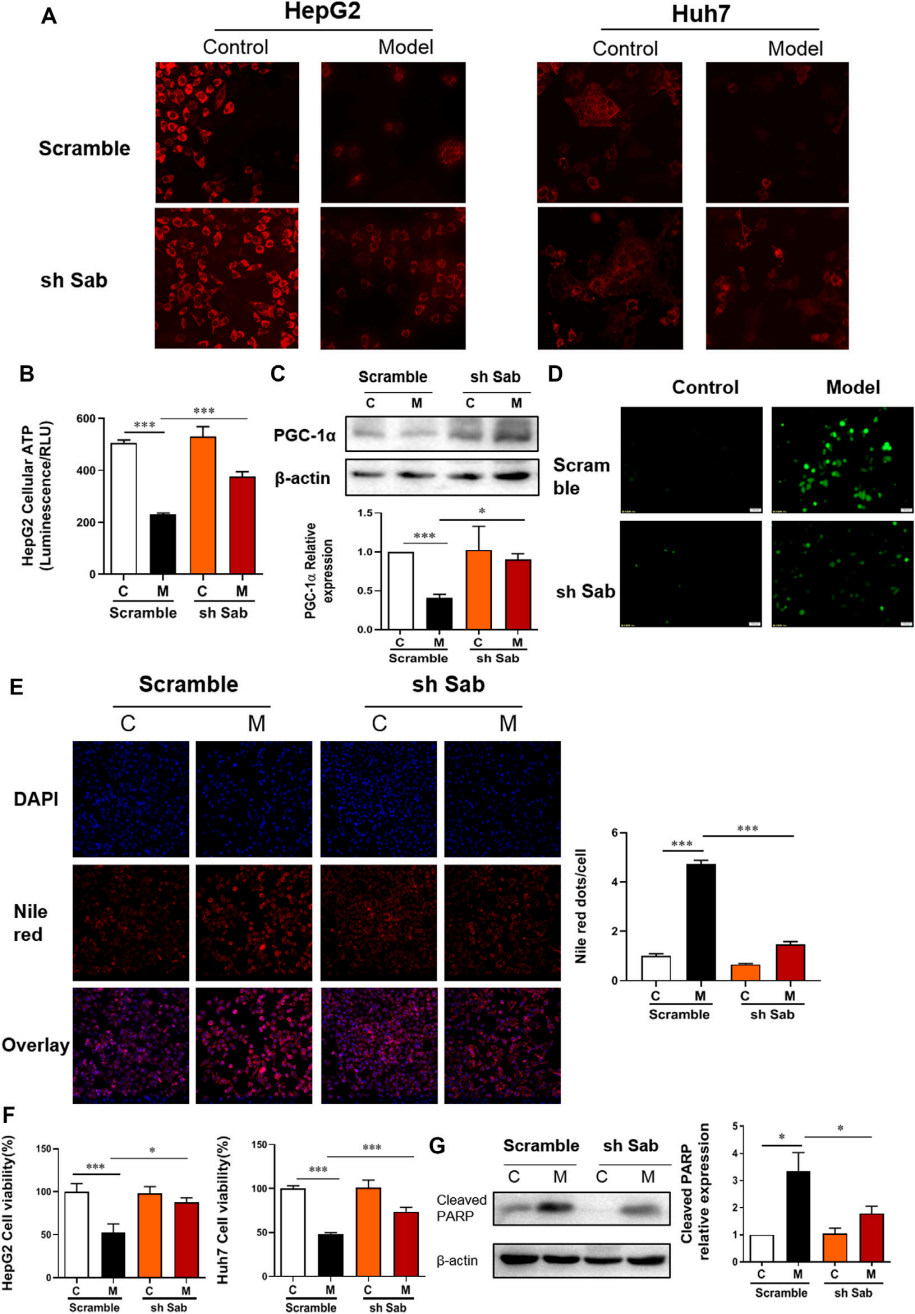
Sab knockdown improved PA-induced mitochondrial dysfunction and lipotoxic injury of hepatocytes. **(A)** TMRM staining of HepG2 and Huh7 cells (200×). **(B)** The ATP production of HepG2 cells. **(C)** PGC-1α protein expression of HepG2 cells. **(D)** ROS level of HepG2 cells (200×). **(E)** DAPI and Nile Red double staining of HepG2 cells (200×). **(F)** The effect of Sab knockdown on HepG2 cell viability with PA induction. **(G)** The effect of Sab knockdown on Cleaved PARP level in HepG2 cells. *p < 0.05, ***p < 0.001.

TMRM can accumulate in normal functional mitochondria and emit fluorescent signals. If the mitochondrial transmembrane potential is damaged, TMRM will diffuse, and fluorescence intensity decreases. As shown in Figure 2A; Supplementary Figure S1B, the fluorescence intensity of TMRM in PA-induced hepatocytes (Model) was significantly reduced, indicating impaired membrane potential and function of mitochondria. Whereas cells with Sab knockdown showed higher fluorescence intensity compared with scramble with PA treatment. Figure 2B also demonstrated the knockdown of Sab rescued mitochondrial function, as sh Sab HepG2 cells generated more ATP with PA-induction when compared with the scramble cells. As a nuclear transcription co-activator, PGC-1α can promote the transcription of target genes related to regulating mitochondrial proliferation, mitochondrial respiration chain, and β-oxidation of fatty acid (Guerrero-Beltrán et al., 2017). Western blot results showed that Sab downregulation reversed the decrease of PGC-1α level induced by PA (Figure 2C). Figure 2D showed that intracellular ROS generation increased notably in the PA-induced hepatocytes. When Sab was knocked down, the green fluorescence was significantly reduced, indicating the inhibitory effect of Sab knockdown on oxidative stress during PA-induced lipotoxic injury.

DAPI and Nile Red double staining was performed to observe cellular lipid accumulation. As shown in Figure 2E and Supplementary Figure S1C, the control HCC cells showed no obvious lipid deposition. After PA incubation for 24 h, Nile red signal was significantly enhanced, whereas the DAPI-presented cell number was reduced, and the Nile red dots normalized to cell number indicated the increased intracellular lipid content. Meanwhile, the decreased number and the presence of some irregular morphology of nuclei showed by DAPI staining also indicated cell damage with PA induction. Sab knockdown alleviated the PA-induced lipid accumulation and the decrease of cell number significantly. Similarly, the CCK8 assay also showed Sab knockdown significantly improved the viability of HepG2 cells and AML12 cells when treated with 0.5 mM PA for 24 h (Figure 2F, Supplementary Figure S1D). PARP protein can be cleaved and activated during apoptosis. Western blot results showed that PA increased the expression of cleaved PARP of scramble HepG2 cells significantly, while the Sab-knockdown cells had a lower expression, suggesting that lipid-induced apoptosis had been alleviated by inhibiting JNK/Sab signaling (Figure 2G).

### Scoparone Reversed PA-Induced Lipotoxic Injury of Hepatocytes

**FIGURE 3:**
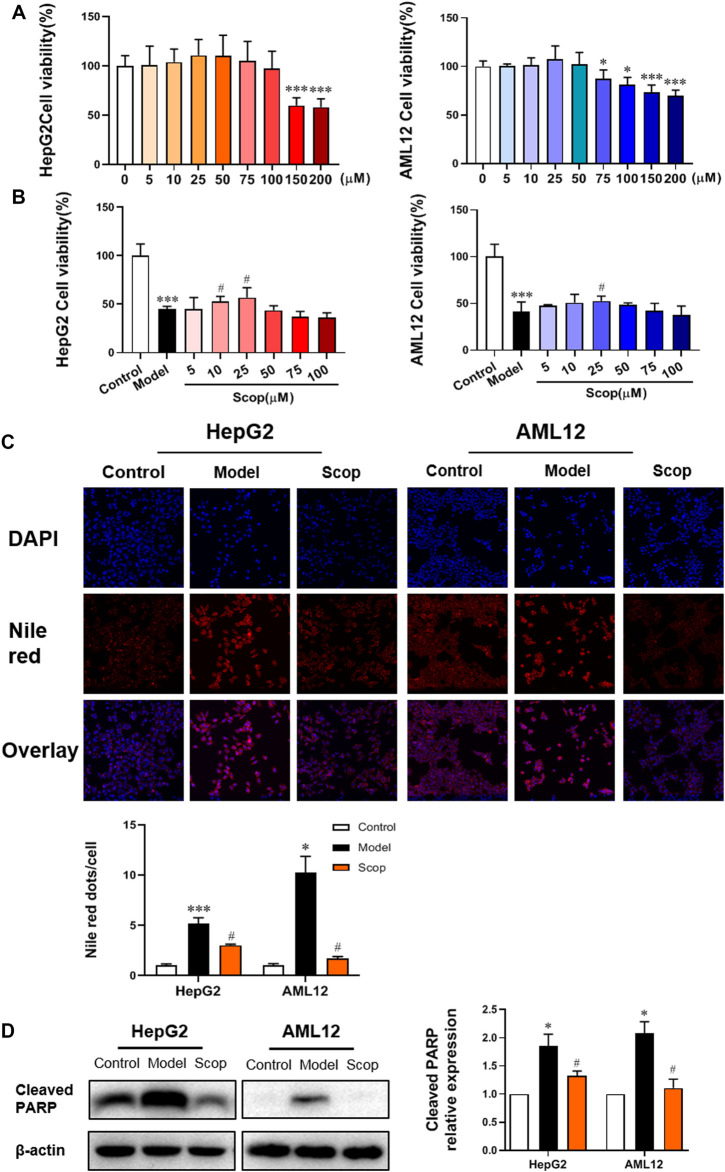
Scoparone reversed PA-induced lipotoxic injury of hepatocytes. **(A)** The effect of different concentrations of Scoparone on the viability of HepG2 and AML12 cells. *p < 0.05, ***p < 0.001 vs. Scop 0 µM. **(B)** The effect of different concentrations of Scoparone on the viability of HepG2 and AML12 cells with PA induction. **(C)** DAPI and Nile Red double staining of HepG2 and AML12 cells. **(D)** The effect of Scoparone on Cleaved PARP protein level in HepG2 and AML12 cells. *p < 0.05, ***p < 0.001 vs. Control; ^#^ *p* < 0.05 vs. Model.

To determine the concentration of scoparone to be used, the viability of HepG2 and AML12 cells incubated with scoparone at different doses was detected by CCK8 assay. Compared with control, cell viability increased with the increase of concentration and reached a peak at 25 µM. The calculated IC50 value of HepG2 cells was 350 µM and that of AML12 cells was 480 µM (Figure 3A). With 0.5 mM PA induction, the viability of HepG2 and AML12 cells decreased significantly. Whereas intervened by scoparone at 10 and 25µM, cell viability was improved, with the highest viability at 25 µM (Figure 3B). The concentration of 25 µM was far less than the IC50 value of scoparone in hepatocytes, which was thus selected to be used in subsequent experiments.

DAPI and Nile red double staining were performed on the hepatocytes in each group. Compared with the model group, the number of cells with 25 µM scoparone treatment group increased significantly and the lipid deposition decreased significantly (Figure 3C). Western blot results presented that scoparone could significantly reduce the expression of apoptosis indicator protein cleaved PARP in HepG2 and AML12 cells induced by PA (Figure 3D).

### PA-Induced Mitochondrial Dysfunction Was Reversed by Scoparone

**FIGURE 4:**
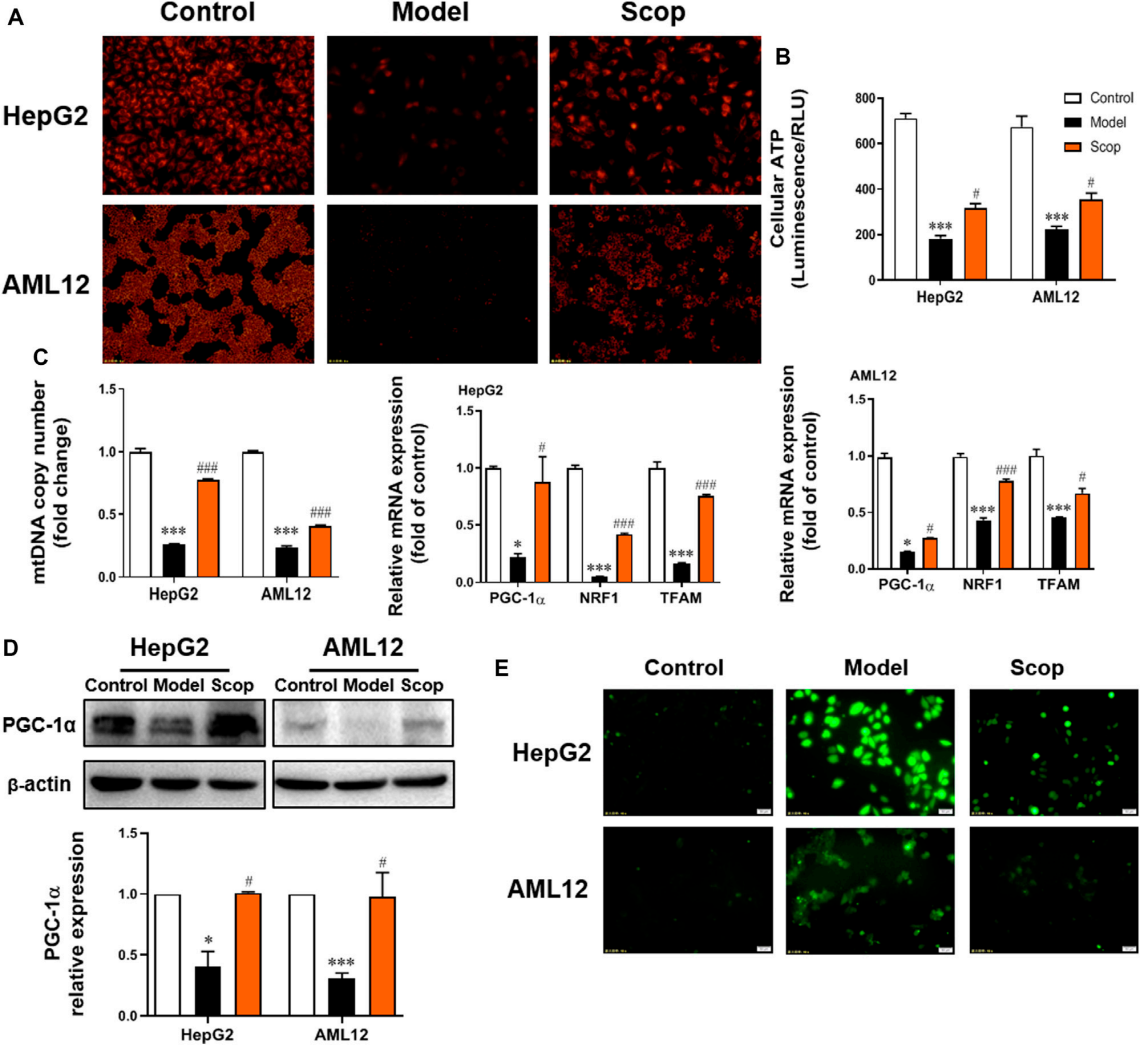
Scoparone improved PA-induced mitochondrial dysfunction in hepatocytes. **(A)** TMRM staining (200×). **(B)** The ATP content of hepatocytes. **(C)** The mRNA level of mtDNA, PGC-1α, NRF1, TFAM in HepG2 and AML12 cells. **(D)** The effect of Scoparone on PGC-1α protein level in HepG2 and AML12 cells. **(E)** ROS staining of HepG2 and AML12 cells (200×). ***p < 0.001 vs. Control; ^#^ *p* < 0.05*,* ^###^ *p* < 0.001 vs. Model.

The TMRM staining found that scoparone treatment could significantly improve the attenuation of cellular mitochondrial membrane potential caused by PA (Figure 4A). As shown in Figure 4B, scoparone could also improve the PA-induced decrease of ATP production. Compared with the control group, the mtDNA copy number, the mRNA levels of PGC-1α, NRF1, and TFAM, as well as PGC-1α protein level were detected downregulated in PA-induced cells, which was improved by scoparone significantly (Figures 4C,D). The ROS level of cells in each group was observed under the fluorescence microscope. Compared with the model group, the ROS generation of cells in scoparone group was significantly reduced and the oxidative stress injury was improved (Figure 4E). Together, these results indicated that the PA-induced damage of mitochondrial function was improved by scoparone.

### Scoparone Inhibited PA-Induced Activation of JNK/Sab Signaling Pathway

**FIGURE 5:**
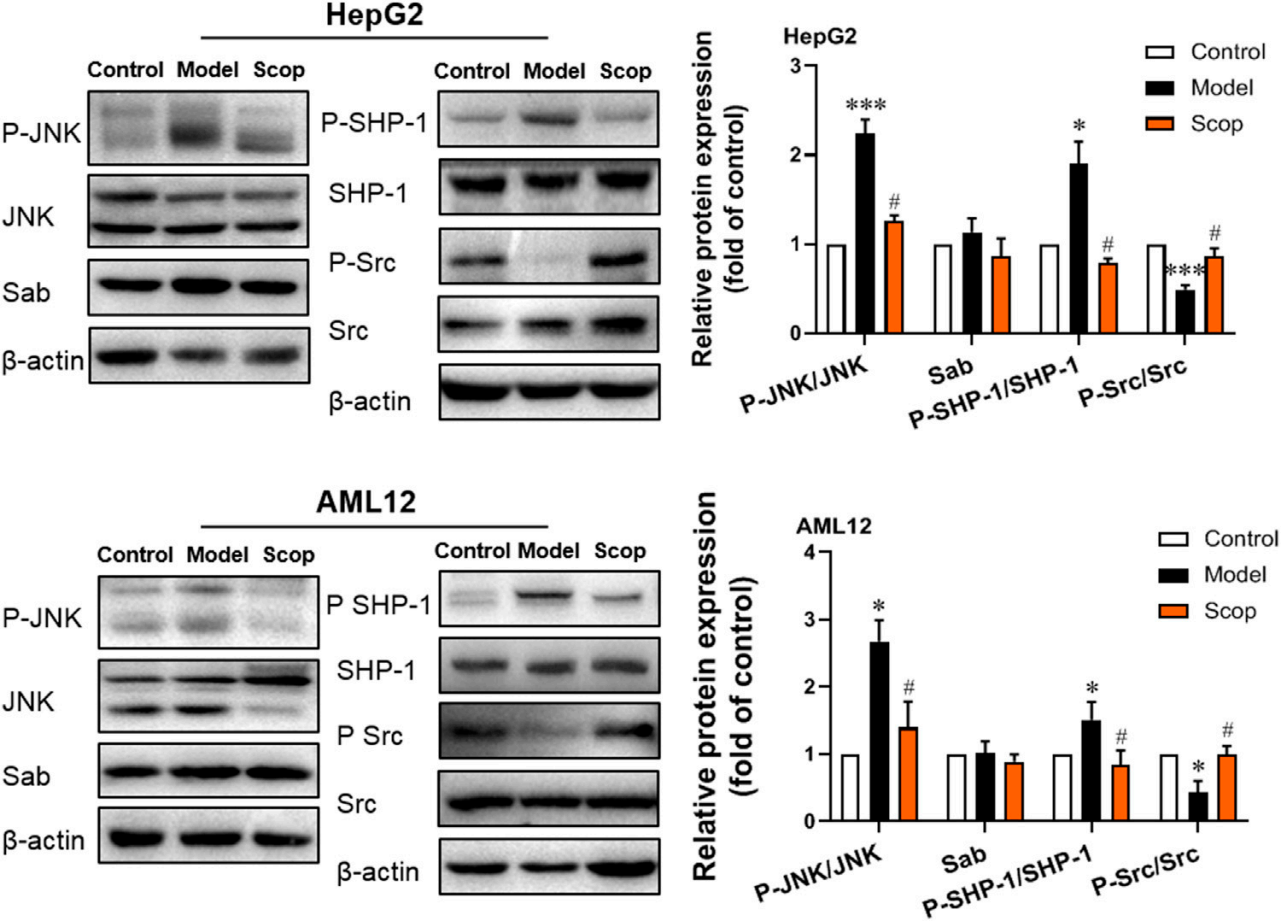
Scoparone inhibited PA-induced activation of JNK/Sab signaling pathway in hepatocytes. The expression and activation levels of JNK, SHP-1, Src, and Sab protein in HepG2 and AML12 cells with PA induction were detected and analyzed. *p < 0.05, ***p < 0.001 vs. Control; ^#^ *p* < 0.05 vs. Model.

The protein expression of JNK/Sab signaling pathway-related molecules in HepG2 and AML12 cells were detected, which were shown in Figure 5. Compared with the control group, the ratio of P-JNK/JNK and P-SHP-1/SHP-1 increased, and the ratio of P-Src/Src decreased significantly. With the intervention of scoparone, compared with the model group, the ratio of P-JNK/JNK and P-SHP-1/SHP-1 decreased, and the ratio of P-Src/Src increased significantly. There was no significant difference in the expression of Sab in cells of different groups.

### Scoparone Alleviated Lipotoxic Liver Injury Induced by HFD in NASH Mice

**FIGURE 6:**
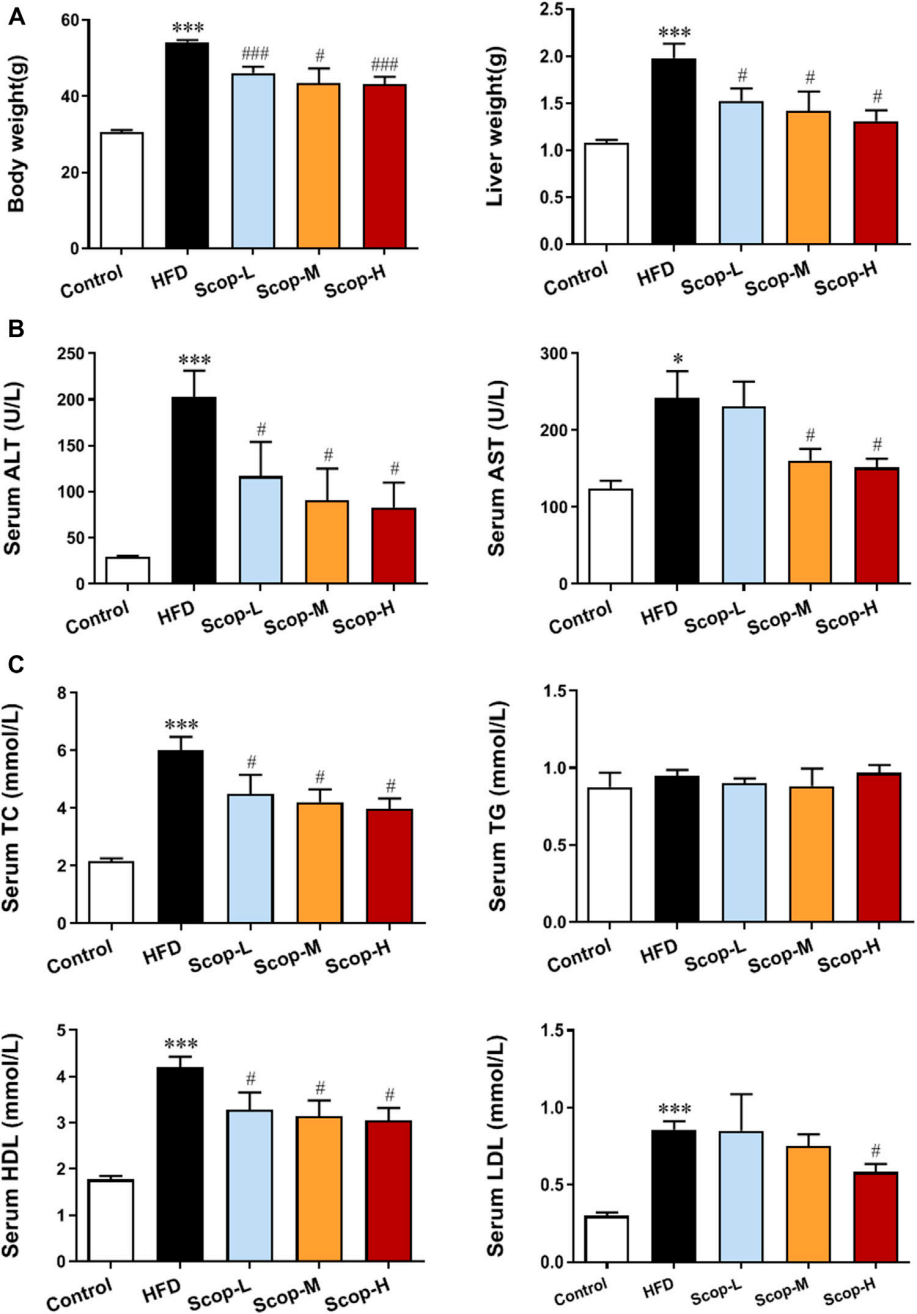
Scoparone improved the body weight, liver weight and serum levels of lipid and aminotransferases in the HFD-induced NASH mice. **(A)** Body weight and liver weight of mice. **(B)** Serum lipid level. **(C)** Serum liver enzyme levels. *p < 0.05, ***p < 0.001 vs. Control; ^#^ *p* < 0.05*,* ^###^ *p* < 0.001 vs. Model.

As shown in Figure 6A, after 30 weeks of the HFD-induction ***,*** the body weight and liver weight of mice in the model group were increased significantly compared with the control group. These were down-regulated by scoparone treatment in a dose-dependent manner. The levels of serum ALT, AST, LDH, TC, HDL, and LDL in HFD-fed mice were higher than the control group, which were also decreased by scoparone dose-dependently (Figures 6B,C).

**FIGURE 7:**
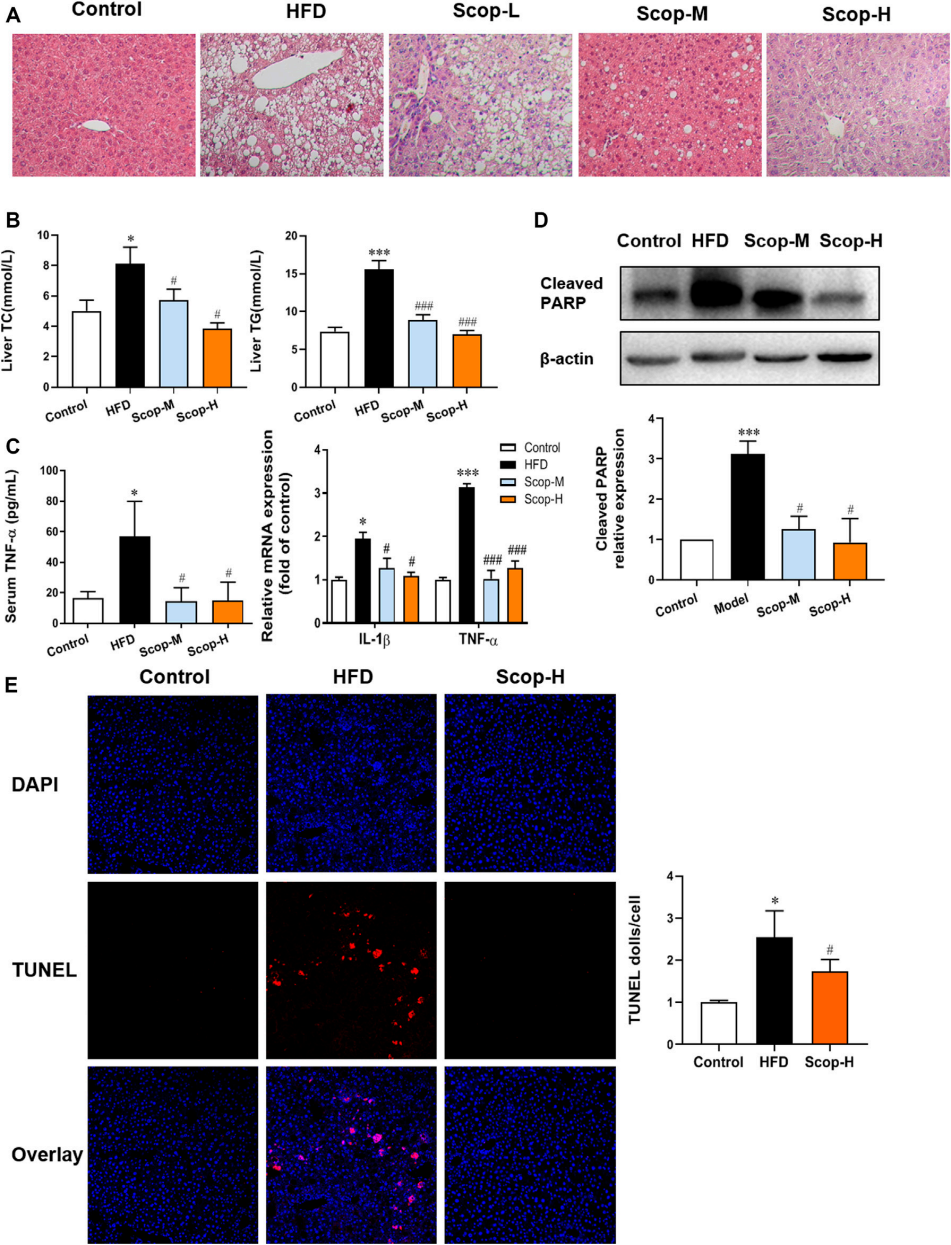
Scoparone reduced the lipotoxic liver injury in the HFD-induced NASH mice. **(A)** HE staining of mouse liver tissues (200×). **(B)** The TG and TC content of mouse liver tissues. **(C)** Serum TNF-α level and IL-1β, TNF-α mRNA expression of liver tissues. **(D)** Cleaved PARP protein expression in mouse liver tissues. **(E)** TUNEL staining of mouse liver tissues (200×). *p < 0.05, ***p < 0.001 vs. Control; ^#^ *p* < 0.05*,* ^###^ *p* < 0.001 vs. Model.

The liver tissues of model mice stained with HE demonstrated the histology characteristics of NASH, with the abundant accumulation of lipid droplets in hepatocytes, scattered lobular inflammatory infiltration, and hepatocellular ballooning. The medium and high dose of scoparone improved the histopathological changes of NASH mice (Figure 7A). The levels of TG and TC in the liver tissues were also reduced by scoparone (Figure 7B), which was consistent with the impaired hepatosteatosis shown in HE staining. Moreover, the levels of serum TNF-α and mRNA expression of IL-1β, TNF-α in the liver tissues of scoparone groups were decreased as compared with NASH mice (Figure 7C). The Western blot results showed the Cleaved PARP protein level in the model group was significantly increased versus control, which was decreased by scoparone treatment (Figure 7D). Figure 7E showed red fluorescence in the nuclei in a large number of cells in the liver tissues of NASH model mice, whereas no obvious positive TUNEL staining in the liver tissues of control and scoparone groups. The quantified fluorescence value normalized to cell number according to DAPI counterstain revealed that the apoptotic rate of liver cells in the model group was significantly higher than that of the control and the scoparone groups.

### Scoparone Alleviated Mitochondrial Dysfunction in the Hepatocytes of NASH Mice by Inhibiting JNK/Sab Signaling Pathway

**FIGURE 8:**
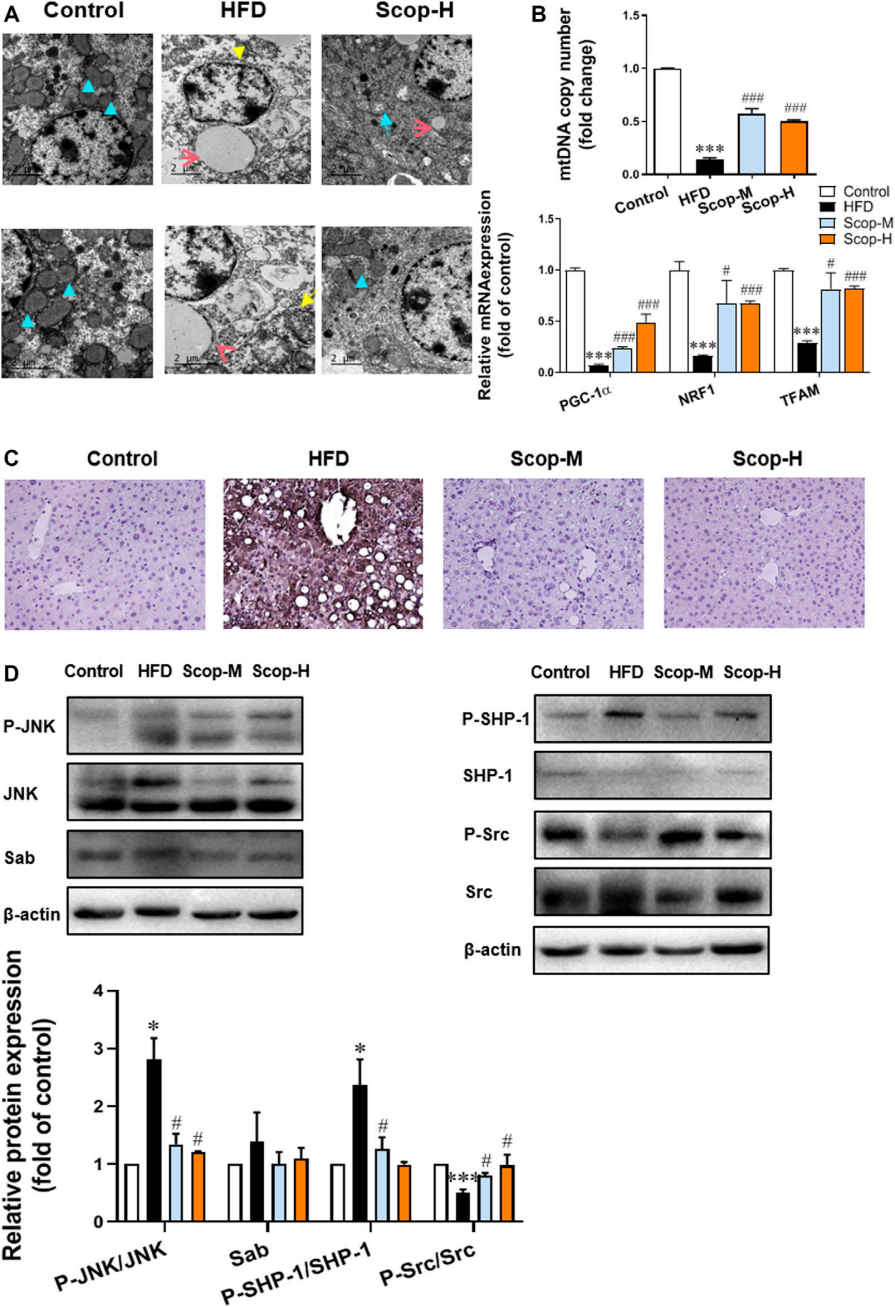
Scoparone inhibited the JNK/Sab signaling pathway and improved mitochondrial dysfunction in the liver tissues of NASH mice. **(A)** Electron microscope images of the mouse liver tissues (blue arrow: normal mitochondria, yellow arrow: mitochondrial cavitation, red arrow: lipid droplets) (200,00×). **(B)** PGC-1α protein expression in mouse liver tissues. **(C)** IHC staining of 4-HNE in mouse liver tissues (200×). **(D)**The expression and/or activation level of JNK/Sab signaling-related protein. ***p < 0.001 vs. Control; ^#^ *p* < 0.05*,* ^###^ *p* < 0.001 vs. Model.

Through the transmission electron microscopy images of liver tissues (Figure 8A), we can observe a closed cystic structure composed of the bilayer membrane of the normal mitochondria. In the slices of the model group, the mitochondrial number was significantly reduced and the mitochondria swelled when compared with control. And as indicated by the yellow arrow, the mitochondria of the model group were cavitated, which may be attributed to reduced matrix particles. While in the scoparone group, there were almost no lipid droplets and the number of mitochondria was more than that of the model group. The mRNA levels of PGC-1α, NRF1, and TFAM, and relative copy number of mtDNA were detected reduced in the liver of NASH mice, which were raised by scoparone significantly (Figure 8B). Mitochondrial dysfunction is generally involved in excessive ROS production, which can trigger lipid peroxidation to cause cell damage. 4-hydroxynonenal (4-HNE), a product of lipid peroxidation, is often used to indicate the ROS level. As shown in Figure 8C, the IHC of 4-HNE displayed an obviously higher level of oxidative stress in the liver tissues of the model group than that in the control, which was improved by scoparone treatment.

The expression levels of JNK/Sab pathway-related molecules in mice liver tissues were also measured (Figure 8D). There was no difference in Sab expression as well as total JNK and Src protein among different groups. Compared with the control, P-JNK/JNK and P-SHP-1/SHP-1 ratios were significantly increased, while P-Src/Src ratio decreased in the liver tissues of NASH mice. Scoparone could reverse the changes of these protein ratios, suggesting its inhibitory effect on the activation of JNK/Sab signaling pathway in the liver of NASH mice.

## Discussion

Lipotoxic liver injury refers to cell damage or even cell death attributed to the excessive accumulation of fatty acids in the liver, which is the most obvious characteristic of NASH (Machado and Diehl, 2016). Mitochondrial dysfunction induced by an overload of fatty acids is regarded as a major factor for lipotoxic liver injury of NASH (Satapati et al., 2012). Mitochondria are organelles with a biolayer membrane composed of four parts: the outer membrane, intermembrane space, inner membrane, and matrix (Vakifahmetoglu-Norberg et al., 2017). Under physiological conditions, mitochondria produce direct energy ATP and a few amounts of ROS to maintain normal cell activities (Shadel and Horvath, 2015). When the mitochondrial function was damaged, electron transfer is blocked and ATP synthesis is suppressed, leading to increased ROS generation (Cortez-Pinto et al., 1999). A high level of ROS, in turn, exacerbates mitochondrial dysfunction and energy dysmetabolism, as well as induces lipid peroxidation to cause cell damage (Kaser et al., 2005; Weltman et al., 2010). In addition, as mitochondrial membrane permeability rises when damaged, apoptosis-related factors release from the mitochondria into the cytoplasm, which could activate downstream proteins of the cysteinyl aspartate specific proteinase (Caspase) family to trigger cell apoptosis (Ma et al., 2014). Moreover, mitochondrial dysfunction can also induce inflammation, necrosis, and fibrosis of liver cells (Ding, 2010). It has been found that the severity of liver steatosis is positively relevant to the degree of mitochondrial function in NASH (Morris et al., 2011). Alleviating mitochondrial damage can reduce lipid accumulation in hepatocytes induced by high glucose (Hsu et al., 2015). And in patients with NASH, liver steatosis can be inhibited by drugs restoring the mitochondrial respiratory chain and reducing inflammation and fibrosis (Begriche et al., 2013).

In this study, the mice induced by a high-fat diet showed diffuse liver steatosis, accompanied by increased apoptosis and up-regulation of transaminase, indicating the lipotoxic liver injury in the NASH model. Of note, we found abnormal mitochondrial structure and decreased quantity in the hepatocytes and decreased PGC-1α protein expression, as well as increased lipid peroxide in the NASH mice. The *in vitro* experiment also showed PA-induced cell apoptosis and lipid accumulation, which resulted in lipotoxic damage. In addition, the decrease of mitochondrial membrane potential, ATP level, and PGC-1α protein expression, and the increase of ROS level indicated mitochondrial dysfunction in NASH mice. Consistent with previous studies, these results also proved the crucial role of lipid-induced mitochondria dysfunction in NASH progress. But the regulated mechanism remains unclear.

It has been found that sustained JNK activation plays an important role in the lipotoxic injury of NASH. JNK can be phosphorylated and activated by a variety of stress-related signals, such as saturated fatty acids, ROS, and pro-inflammatory cytokine (Gan et al., 2014). Its activation could upregulate the transcription of inflammatory cytokines and promote their release through activating the NLR family pyrin domain-containing protein 3 (NLRP3) inflammasome (Li et al., 2015). JNK can also mediate cell apoptosis by inducing activation of transcription factors like c-Jun to produce apoptotic related molecules or by directly regulating the activation of Bcl-2 family members (Han et al., 2009; Win et al., 2011). It was reported that continuous JNK activation could damage the function of the mitochondrial respiratory chain and causes mitochondrial dysfunction (Gan et al., 2014). Furthermore, previous studies have found that JNK is activated in a Sab-dependent manner in acetaminophen (APAP) and tumor necrosis factor/Galactosamine-induced acute liver injury of mice (Win et al., 2016). Under physiological conditions, the mitochondrial membrane protein Sab binds to SH2-containing protein tyrosine phosphatase (SHP-1). Whereas under stress pathological state during liver injury, continuously activated JNK specifically binds to Sab and causes its conformational change, which makes SHP-1 separate from Sab and is phosphorylated by non-receptor tyrosine kinase c-Src (Tyrosine Phosphatase c-Src, Src). SHP-1 is an important member of the protein tyrosine kinase (PTK) family and plays a role in maintaining the phosphorylation level of tyrosine (Zhang et al., 2000). Src is located both on the outer membrane and inside the mitochondrion (Gomez-Puerta and Mocsai, 2013). It regulates multiple signal transduction involving processes such as proliferation, differentiation, and apoptosis. Inhibition of Src could directly suppress the electron transport of mitochondria and promote ROS generation (Yeatman and Timothy, 2004; Sharma et al., 2016). In turn, excess ROS can reduce ATP production and the activity of Src (Srikanthan et al., 2016). Activated SHP-1 by JNK/Sab is transferred to the inner mitochondrial membrane and mediates the inactivation of Src, thereby causing mitochondrial dysfunction (Win et al., 2015; Win et al., 2016).

**FIGURE 9:**
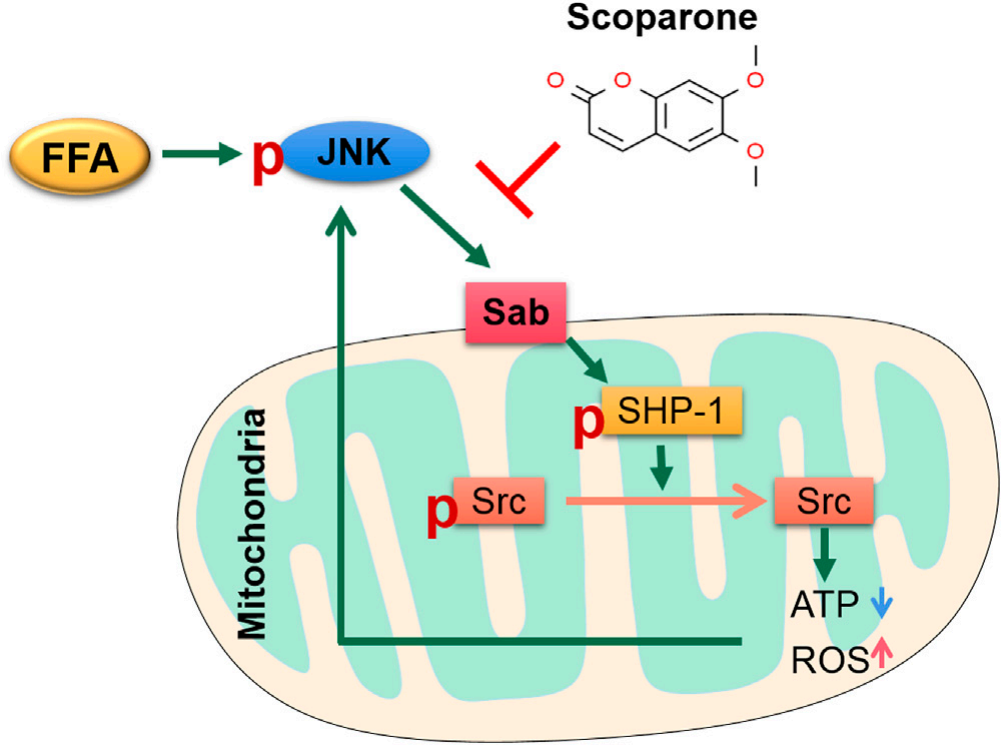
The sketch of the mechanism of JNK/Sab signaling pathway involved in mitochondrial dysfunction in NASH and the effect of Scoparone. Stimulated by free fatty acids, JNK is activated and binds to the outer mitochondrial transmembrane protein Sab, which then releases and promotes the activation of SHP-1 in mitochondria. Phosphorylated SHP-1 could induce the inactivation of Src, which leads to damage of mitochondrial electron transport chain, thereby reducing ATP production and increasing ROS generation. The mitochondrial damage in turn stimulates JNK and thus results in sustained activation of JNK. Scoparone can reverse this process partly through inhibiting the JNK/Sab signaling.

In this study, to further clarify the role of the JNK/Sab pathway in NASH-related hepatotoxicity injury, we used the key molecule Sab knockdown cells in PA induction experiments. It was found that, compared with the scramble, Sab knockdown inhibited the activation of downstream SHP-1 while increasing Src phosphorylation level, suggesting that Sab knockdown has an inhibitory effect on this pathway. Affected by the inhibition of the JNK/Sab pathway, the mitochondrial dysfunction of Sab knockdown cells was improved than that of scramble cells induced by PA, as evidenced by the restorative upregulation of mitochondrial ATP production and mitochondrial membrane potential. And in the hepatocytes with Sab knockdown, cell viability was enhanced, whereas lipid accumulation and apoptosis were reduced, when compared with scramble cells. Interestingly, Sab knockdown also significantly reduced the phosphorylated JNK level, probably because that Sab knockdown reversed PA-induced mitochondrial dysfunction and reduced ROS level, forming a feedback inhibition to JNK. Collectively, our results indicate that JNK/Sab signaling pathway is activated after fatty acids deposit in hepatocytes of NASH mice, a mechanism that may be involved in liver mitochondrial dysfunction and liver damage (Figure 9).

At present, TCM is widely used in the treatment of fatty liver disease and shows curative effects. Based on the pathogenesis of NAFLD of TCM theory, we developed the compound formula Jiangzhi Granule, composed of five herbal medicine including Artemisiae Scopariae Herba, which is effective for the clinical treatment of NAFLD (Pan et al., 2013). Previous studies have shown that the compound can improve hepatic steatosis and inflammation, reduce serum transaminases, and inhibit JNK activation in liver tissue of NASH mice (Liu et al., 2014). To further explore its mechanism, pharmacological research on its components was carried out. Scoparone, one component of Artemisiae Scopariae Herba, was found effective in reducing lipid accumulation and protecting liver function. The *in vivo* experiments in our study confirmed its potential against lipotoxic liver injury in the HFD-induced NASH mice. This effect against liver injury is consistent with some previous studies. It has been found that scoparone could improve acute liver injury in mice caused by p-acetylphenol, significantly reducing histopathological changes in liver tissue and cell apoptosis (Hui Y. et al., 2020). In an *in vivo* experiment of cholestatic liver disease, scoparone inhibited liver inflammation and fibrosis, improved cholestasis, and promoted the recovery of liver injury by upregulating the expression of farnesoid X receptor/bile salt export pump (FXR/BSEP) pathway (Yuan et al., 2020). Liu X Showed that scoparone could inhibit the transforming growth factor-β (TGF-β)/Smad/Stat3 signaling pathway, therefore inhibiting the proliferation of hepatic stellate cells and significantly reducing liver fibrosis (Liu and Zhao, 2017).

Notably, Liu B et al. have shown that scoparone could improve hepatocyte inflammation, apoptosis, inflammation as well as hepatic steatosis in methionine and choline-deficient (MCD) diet-induced NASH mice, which was similar to our results in HFD-induced mice. However, in their *in vitro* experiment, scoparone was found unable to reduce the hepatosteatosis in PA-induced AML12 cells. And it was concluded that the effect of scoparone against NASH through blocking TLR-4/NF-κB signaling-mediated immune responses of macrophages (Liu et al., 2020). Different from the above study, our results demonstrated that scoparone could reverse PA-induced lipotoxic injury of hepatocytes, such as cell viability, apoptosis, and lipid accumulation in HepG2 and AML12 cells. This contradiction may be due to the different intervention conditions and the degree of cell damage in the two experiments, which may need further analysis and confirmation. However, scoparone has been shown capable of regulating lipid metabolism in some previous researches. In one study, scoparone exposure at a low, non-cytotoxic dose significantly altered metabolism in primary hepatocytes isolated from ICR male mice. Lipid changes, e.g., the levels of identified PG (19:1 (9Z)/14:0), PE (17:1 (9Z)/0:0), PE (19:1 (9Z)/0:0) were found to be upregulated in the ethanol-induced group, which were downregulated in scoparone group (Zhang et al., 2016). Moreover, scoparone could inhibit TG accumulation in the mature adipocytes. Further study revealed that scoparone negatively regulated the expression level and transcriptional activity of the key adipogenic transcription factor, PPARγ, in 3T3-L1 preadipocytes and suppressed adipogenesis (Noh et al., 2013). These studies support that scoparone could suppress the lipotoxic injury of hepatocytes directly.

In ethanol-induced HepG2 cells, it has been found that scoparone could ameliorate oxidative stress-mediated injury (Noh et al., 2011). Our results also demonstrated the level of ROS and lipid peroxide 4-HNE was downregulated by scoparone. Moreover, PA-induced reduction of mitochondrial membrane potential and ATP generation of hepatocytes, as well as the changes of mitochondrial number and morphology in the liver tissues of NASH mice were reversed by scoparone treatment, indicating the effect of scoparone in improving mitochondrial number and function. As mitochondrial dysfunction is the major contributor to ROS generation, thus the anti-oxidative stress role of scoparone might be through restoring mitochondrial function. Further study showed that scoparone treatment could reverse the PA-induced activation of JNK and SHP-1, and inactivation of Src in the experiments *in vitro* and *in vivo*, which was consistent with the results of Sab knockdown. As the aforementioned involvement of JNK/Sab signaling in mitochondrial dysfunction of hepatocytes, we consider that scoparone could ameliorate the lipotoxic liver injury in NASH partially via inhibiting the JNK/Sab pathway and improving mitochondrial dysfunction (Figure 9).

## Conclusion

In summary, the accumulation of fatty acids activates JNK/Sab signaling pathway to induce mitochondrial dysfunction, promoting ROS release and cell apoptosis, which contributes to the lipotoxic liver injury in NASH. Blocking this signaling pathway can reverse hepatic steatosis and cell damage. Moreover, we confirmed the effect of the naturally-derived compound scoparone against HFD-induced NASH. The pharmacological mechanism of scoparone involves the inhibition of JNK/Sab signaling-mediated mitochondrial dysfunction. Therefore, scoparone may serve as a potential therapeutic compound in the treatment of NASH.

## Data Availability

The original contributions presented in the study are included in the article/Supplementary Material, further inquiries can be directed to the corresponding authors.
