# Supplementary material for: Scoparone Improves Nonalcoholic Steatohepatitis Through Alleviating JNK/Sab Signaling Pathway-Mediated Mitochondrial Dysfunction
Source: Front Pharmacol. 2022 May 3;13:863756. doi: 10.3389/fphar.2022.863756 (PMC9110978; doi:10.3389/fphar.2022.863756)
Supplement: Supplementary file 1 [file DataSheet1.docx]

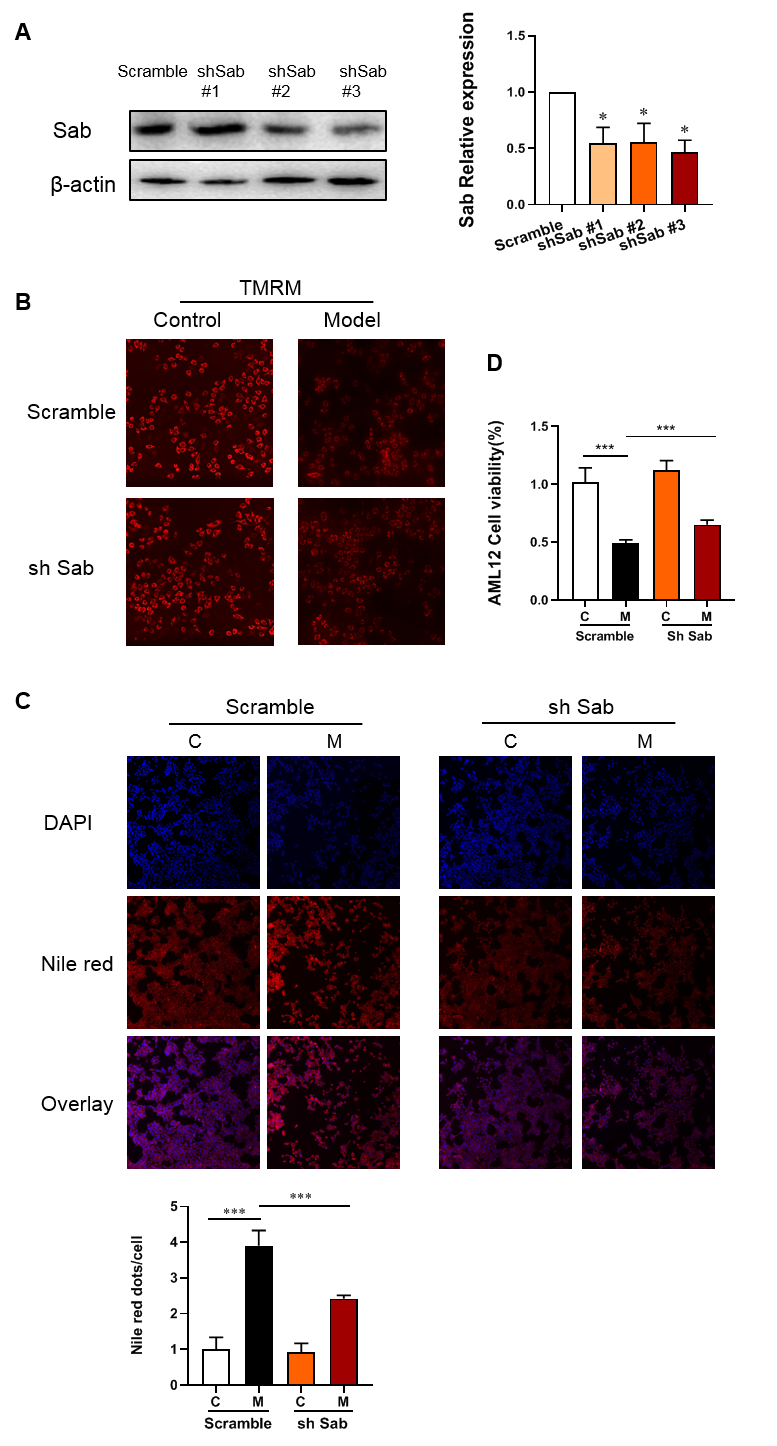


**Supplementary Fig.1** Sab knockdown improved PA-induced mitochondrial dysfunction and lipotoxic injury of AML-12 cells. (A) The protein expression level of Sab in AML-12 cells transfected with Sab shRNA lentivirus or scramble. **P* < 0.05 vs. Scramble. (B) TMRM staining of cells (200×). (C) DAPI and Nile Red double staining of AML-12 cells (100×). (D) The effect of Sab knockdown on AML-12 cell viability with PA induction. ****P* <0.001.
